# Intestinal Schistosomiasis in Mothers and Young Children in Uganda: Investigation of Field-Applicable Markers of Bowel Morbidity

**DOI:** 10.4269/ajtmh.2010.10-0307

**Published:** 2010-11-05

**Authors:** Martha Betson, Jose Carlos Sousa-Figueiredo, Candia Rowell, Narcis B. Kabatereine, J. Russell Stothard

**Affiliations:** Wolfson Wellcome Biomedical Laboratories, Department of Zoology, Natural History Museum, London, United Kingdom; Department of Infectious and Tropical Diseases, London School of Hygiene and Tropical Medicine, London, United Kingdom; Vector Control Division, Ministry of Health, Kampala, Uganda

## Abstract

To control intestinal schistosomiasis at a national level in sub-Saharan Africa, there is a need for field-applicable markers to measure morbidity associated with this disease. The purpose of this study was to determine whether fecal calprotectin or fecal occult blood assays could be used as morbidity indicators for intestinal schistosomiasis. The study was carried out in Uganda with a cohort of young children (n = 1,327) and their mothers (n = 726). The prevalence of egg-patent schistosomiasis was 27.2% in children and 47.6% in mothers. No association was found between schistosomiasis infection and fecal calprotectin in children (n = 83, odds ratio [OR] = 1.08, *P* = 0.881), although an inverse relationship (n = 58, OR = 0.17, *P* = 0.043) was found in mothers. Fecal occult blood was strongly associated with *Schistosoma mansoni* infection in children (n = 814, OR = 2.30, *P* < 0.0001) and mothers (n = 448, OR = 1.95, *P* = 0.004). Fecal occult blood appears to be useful for measuring morbidity associated with intestinal schistosomiasis and could be used in assessing the impact of control programs upon disease.

## Introduction

Schistosomiasis (bilharzia) is a neglected tropical disease caused by trematode parasitic worms of the genus *Schistosoma*. Approximately 207 million persons are infected worldwide,^1^ which leads to the loss of approximately 1.53 million disability-adjusted life years.^2^ The greatest burden of disease is found in sub-Saharan Africa,^3^ where intestinal schistosomiasis, caused by infection with *Schistosoma mansoni* and to a lesser extent by *S. intercalatum* or *S. guineensis*, occurs.

Pathologic changes associated with intestinal schistosomiasis are predominately brought about by detrimental immunologic responses to eggs, produced by female worms, which become trapped in host tissues. Migration of eggs through the intestinal wall, for example, causes perforations and stimulates an initial eosinophilic inflammatory reaction in the mucosa. Granulomas eventually form around the eggs if they are not voided and fibrosis ensues to form large non-malignant masses often known as bilharziomas.^4^–^7^ Common symptoms and pathologic changes range from anemia, abdominal pain, and diarrhea with or without blood, to pseudopolyps, microulcerations, and obstruction of the colon.^2^,^5^,^8^–^12^ Frequently eggs pass by the portal vein into the liver and become lodged in the periportal spaces, which leads to local inflammatory reactions that can result in hepato-splenomegaly, portal hypertension, and gastrointestinal varices.^2^,^4^,^8^,^13^,^14^ Ultimately bleeding from esophageal varices may occur, which can be fatal with hematemesis as an obvious visual sign.^15^

National programs to control schistosomiasis have been conducted in Brazil and China for more than 20 years but have only recently been established in sub-Saharan Africa. These programs are based on regular mass distribution of the anthelminthic drug praziquantel, and their main aim is the control of schistosomiasis-associated morbidity rather than infections *per se*.^16^ School-aged children have been particularly targeted for mass chemotherapy because they generally show the highest infection intensities (as assessed by egg counts in stool) and were thought to be the group most likely to respond to praziquantel treatment with subsequent reductions in morbidity.^17^ However, recent work has indicated that pre-school children are also at risk of infection and can benefit from chemotherapy.^18^,^19^

The repertoire of simple, inexpensive, and non-invasive methods to measure the intestinal morbidity associated with schistosomiasis is rather limited, and new assays are urgently needed to evaluate the impact of control programs (through assessment of morbidity pre-treatment and post-treatment) and to determine the overall disease burden associated with schistosomiasis.^11^,^15^,^20^ Clinical signs and symptoms such as anemia, abdominal pain, and diarrhea are not necessarily specific to schistosomiasis but associations can be strong in high-transmission areas.^21^ Intestinal granulomas, fibrosis, and ulceration are only detectable through invasive methods such as rectal biopsy or sigmoidoscopy, which require hospitalization and cannot be applied on a large scale or in the field.^4^,^20^

Recent work indicates that fecal levels of eosinophil cationic protein and eosinophil protein X may prove to be useful markers for intestinal morbidity. However, the assay is relatively complex and requires freezing of stool extracts and transport to a well-equipped laboratory.^22^ In contrast, a number of field-applicable tools exist for assessment for hepatic and splenic pathology, which develop later in the progression of the disease. These tools include clinical palpation of the liver and spleen and ultrasonography by using portable ultrasound machines.^14^,^20^ However, the search for direct markers of bowel morbidity continues.

Calprotectin, a multimeric complex of the calcium-binding proteins MRP8/S100A8 and MRP14/S100A9, forms around 60% of the cytosolic protein in neutrophil granulocytes and is also found in monocytes and the early differentiation stages of macrophages.^23^ MRP8/S100A8 and MRP14/S100A9 are thought to play a role in the innate immune response and to function as damage-associated molecular patterns. Fecal calprotectin is typically an excellent marker of intestinal inflammation, likely because inflammation is accompanied by increased translocation of calprotectin-containing granulocytes into the intestinal mucosa and thus secretion of calprotectin into the gut lumen.^24^ A number of studies have indicated that determination of fecal calprotectin levels can aid in diagnosis of inflammatory bowel disease and that there is a correlation between fecal calprotectin levels and the degree of inflammatory bowel disease clinical activity.^24^,^25^ It is possible that the lesions and sequelae induced in intestinal schistosomiasis may increase fecal calprotectin levels, for example when eggs perforate the intestinal lining or stimulate inflammatory reactions but this remains to be proven.

Blood in stool is an obvious general marker of intestinal morbidity. Even small amounts of blood in the feces, known as fecal occult blood (FOB), can be indicative of intestinal pathologic changes, for example, colorectal cancer. This finding has led to the development of several point-of-care rapid tests for FOB, and a recent Cochrane review reports that screening by using FOB tests can lead to a modest reduction in colorectal cancer–associated mortality because positive cases can be detected in a more timely fashion.^26^ Despite the widespread availability of FOB tests and the fact that *S. mansoni* eggs perforate the intestinal muscosa and cause a small release of blood into the bowel, few studies have investigated whether FOB can be used as a direct marker of schistosomiasis morbidity. Nevertheless, there is some evidence to suggest that there is an association between schistosomiasis infection and FOB, although tests used show differing sensitivities and recent developments in FOB tests have increased detection limits.^27^,^28^

In this study, we determined whether fecal calprotectin levels or FOB are associated with intestinal schistosomiasis infection and if they could be used in the field as indicators of morbidity. To assess this possibility, we screened a population of young children (early stage pathology) and mothers (late stage pathology) living in lakeshore communities in Uganda endemic for *S. mansoni* infection.

## Methods

### Study participants and treatment.

**Figure 1.**
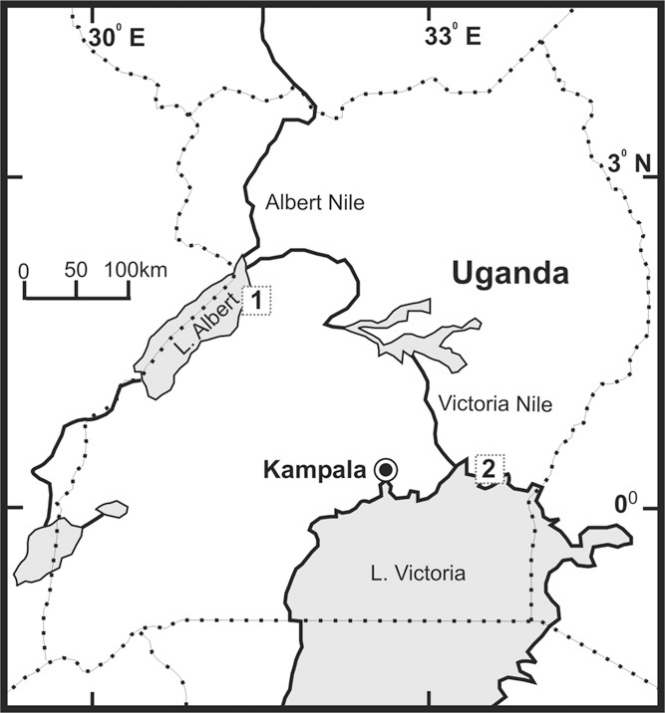
Locations of the study sites in Uganda. 1 = location of the surveyed villages in Buliisa District on Lake Albert, and 2 = location of the villages in Mayuge District on Lake Victoria.

**Figure 2.**
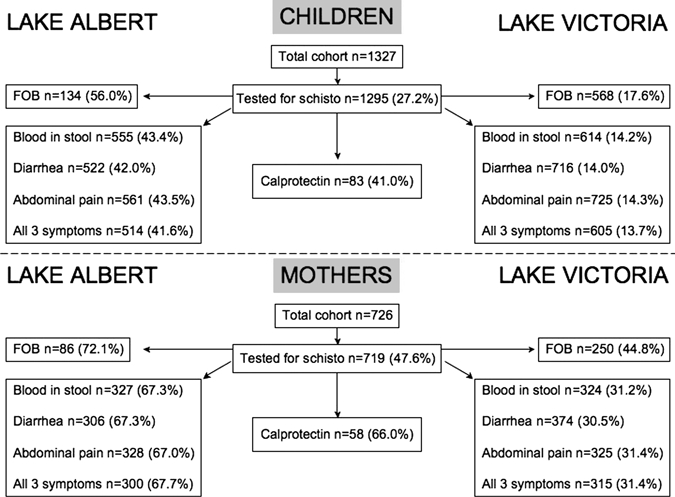
Numbers of children and mothers tested for fecal occult blood and calprotectin, and questioned about blood in stool, diarrhea, and abdominal pain on Lakes Albert and Victoria in Uganda. The percentages of those examined who were positive for *Schistosoma mansoni* are shown in parentheses. Calprotectin analysis was not carried out separately for each lake system; only total numbers of mothers and children tested are shown.

This study was carried out as part of the Schistosomiasis in Mothers and Infants project, a cohort study that is ongoing in six lakeshore communities in Uganda to investigate the infection dynamics of *S. mansoni* and develop better control strategies for younger children. The main cohort consists of 333 mothers and 572 young children who live in three villages in Buliisa District on Lake Albert (Bugoigo, Walukuba, and Piida) and 333 mothers and 639 young children from three villages in Mayuge District on Lake Victoria (Bugoto, Bukoba, and Lwanika). The data used for this study were obtained during baseline epidemiologic surveys in April and June 2009. Data were also included from a pilot study involving 60 mothers and 116 young children living in Kayanja and Walumbe in Mayuge District on Lake Victoria.^19^ Location of study districts are shown in Figure 1. Numbers of children and mothers questioned and tested for fecal occult blood and calprotectin on Lakes Albert and Victoria are shown in Figure 2.

At baseline all participants were treated with praziquantel (for schistosomiasis) and albendazole (for soil-transmitted helminths). For younger/smaller children (less than two years of age), praziquantel tablets were crushed and mixed with orange juice and sugar before administration by spoon-feeding. They also received a chewable orange flavored half-tablet of albendazole.

### Questionnaires.

Each mother was interviewed were interviewed in the local language by a field assistant to ascertain whether she or her children had any history of blood in stool, diarrhea, or abdominal pain. Results were used to generate binary variables (negative or positive responses). These variables were combined to produce one new binary variable (blood in stool and diarrhea and abdominal pain). Subsequently, the questionnaire results were combined with the FOB results to generate four additional variables.

### Human calprotectin enzyme-linked immunosorbent assay.

The human calprotectin enzyme-linked immunosorbent assay (ELISA) was conducted in Kayanja and Walumbe (Mayuge District) and Bugoigo (Buliisa District). In Kayanja and Walumbe, all available stool samples, which there was to time process, were tested. In Bugoigo, a random selection of egg-positive and egg-negative stool samples were tested. Overall, 72 egg-negative and 69 egg-positive stool samples were successfully tested.

Stool samples (one sample per person) were processed in the field within 24 hours of collection. Stool was passed through a 212-μm sieve, and 100 mg of sieved stool was homogenized in 5 mL of extraction buffer (0.1 M Tris, 0.15 M NaCl, 1.0 M urea, 10 mM CaCl_2_, 0.1 M citric acid monohydrate, and 5 g/L of bovine serum albumin). Samples were centrifuged at 10,000 × *g* for 20 minutes, and supernatants were then used in the ELISA, which was carried out by using the Human Calprotectin ELISA Test Kit (Hycult Biotechnology B.V., Uden, The Netherlands) according to the manufacturer's instructions.

Absorbance was read at 450 nm by using an LT-4000 microplate reader (Labtech International Ltd., East Sussex, United Kingdom), and mean absorbance was calculated for each set of duplicate standards, samples, and negative controls. Using Logger Pro version 3.8.2 software (Vernier Software and Technology, Beaverton, OR), we generated a standard curve for each set of standards. This curve was used to determine the concentration of calprotectin in each stool sample, taking into account the relevant dilution factor. Using the recommended dilutions of stool samples, we determined that the minimum concentration of calprotectin that could be detected in stool with this assay is 65 μg/g. The calprotectin concentration data were categorized to produce a binary variable (calprotectin negative and calprotectin positive).

### Fecal occult blood tests.

The FOB tests were carried out on a random sample (because of a limited supply of tests) of persons in the cohort by using the Instalert One Step Fecal Occult Blood Test Device (Innovacon, Inc., San Diego, CA) according to the manufacturer's instructions. One fresh stool sample was tested per person. The specimen collection stick was stabbed into the stool sample at three sites. The stick was placed into a specimen collection tube, the cap was screwed on tightly, and the tube was shaken vigorously to mix the sample and extraction buffer. Two drops of the homogenate were then transferred to the specimen well of the FOB test device. Results were read after five minutes. Results were classified as negative (–), trace, weak positive (+), medium positive (++), and strong positive (+++).

### Detection of intestinal schistosomiasis and soil-transmitted helminths.

Parasitologic diagnosis of *S. mansoni* and soil-transmitted helminths (*Trichuris trichiura*, *Ascaris lumbricoides*, and hookworms) was performed in the field by using two stool samples collected on consecutive days and double Kato-Katz thick smears (2 × 41.7 mg of stools) for each mother and child. Samples were inspected by microscopy.^29^ Results were expressed as mean egg count per gram (epg) of feces. The *S. mansoni* results were categorized as light (< 100 epg), medium (100–400 epg), and high (> 400 epg) intensity. Similarly, the hookworm, *T*. *trichiura*, and *A*. *lumbricoides* infection intensities were classified according to World Health Organization recommendations.^30^

### Statistical analysis.

Data were entered into a spreadsheet by using Microsoft (Redmond, WA) Excel 2004 for Mac (version 11.5.6) and were analyzed by using Stata version 11.0 (StatCorp, College Station, TX) and R version 2.8.1 (http://cran.r-project.org/bin/windows/base/). Age data were categorized for children (< 2, 2–4, > 4 years of age) and mothers (< 25, 25–35, and > 35 years of age). For schistosomiasis infection intensity values, the geometric mean of Willams (GM_W_) and the arithmetic mean of positive samples (AM_POS_) were chosen as the measures of central tendency because of the over-dispersion present in the data. Ninety-five percent confidence intervals (CIs) for GM_W_ were calculated according to the procedure of Kirkwood and Sterne.^31^

To determine whether there was an association between *S. mansoni* infection and the presence of calprotectin in stool, logistic regression was carried out by using the calprotectin binary variable as the response variable and schistosomiasis infection as the explanatory variable. Logistic regression analysis could not be carried out when cut-offs of 100 μg/g or 150 μg/g were used because there were small numbers in some categories. Therefore, the Fisher exact test was used to assess associations.^32^

For analysis of the association between FOB and schistosomiasis infection, FOB results were reclassified as a binary variable in which negative and trace results were combined into one category (negative) and all positive results were combined into the other category (positive). The FOB binary variable was used as the response variable and schistosomiasis infection (either as a binary variable or categorized as light, medium, and heavy infection) was used as the explanatory variable in logistic regression analysis.

To assess associations between a history of blood in stool, diarrhea or abdominal pain (and the combination of these) with schistosomiasis infection, logistic regression analysis was carried out by using the questionnaire variables as the response variables and schistosomiasis infection as the explanatory variable. Similar analysis was carried out to determine whether there was an association between the combined FOB/questionnaire variables and schistosomiasis infection.

During the analysis of FOB, calprotectin, and questionnaire data, children and mothers were considered separately. Additionally, stepwise analysis was performed to assess the effect of potential confounders including village, age, sex, and hookworm infection on all models, and comparison between models was performed by using likelihood ratio tests.

### Ethical approval and informed consent.

The London School of Hygiene and Tropical Medicine, London, United Kingdom (application no. LSHTM 5538·09) and the Ugandan National Council of Science and Technology granted ethical approval for this study. All participating mothers gave informed consent in writing or by fingerprint (in cases of illiteracy) on behalf of themselves and their children.

## Results

The mean age of the children was 3.0 years (age range = 4 months to 6.5 years) and that of the mothers was 28.9 years (age range = 15–70 years). In children, the female to male ratio was 0.95. The overall *S. mansoni* infection prevalence levels were 27.2% in children (GM_W_ = 1.90, 95% CI = 1.61–2.21; AM_POS_ = 47.62 epg, 95% CI = 40.37–56.17; maximum value = 5,749 epg) and 47.6% in mothers (GM_W_ = 6.24, 95% CI = 5.11–7.58; AM_POS_ = 188.88 epg, 95% CI = 147.78–229.98; maximum value = 3,537 epg). *Ascaris lumbricoides* and *T*. *trichiura* infection prevalence levels were low, and 9.9% of children and 32.6% of mothers were infected with hookworms (Table 1).

### Self-reported symptoms.

Overall, 43.7% of children and 46.9% of mothers reported a history of blood in stool, 78.7% of children and 73.4% of mothers reported a history of diarrhea, and 70.5% of children and 79.6% of mothers reported abdominal pain. In children, there was evidence for an association between intestinal schistosomiasis infection and any symptom/combination of symptoms apart from abdominal pain on its own (Table 2). In contrast, there was no association between schistosomiasis and any of the reported symptoms in mothers.

### Calprotectin ELISA.

The calprotectin ELISA was conducted with fresh stool samples from 58 mothers and 83 children. In this subset of mothers and children, the prevalence levels of schistosomiasis were 66.0% and 41.0%, respectively. Overall 26.5% of children and 13.8% of mothers had detectable calprotectin (> 65 μg/g) in their stool. The maximum fecal calprotectin concentration was 812 μg/g in children and 367 μg/g in mothers. There was little evidence for an association between a calprotectin-positive result and schistosomiasis infection in children (odds ratio [OR] = 1.08, 95% CI = 0.40–2.95, *P* = 0.881). In mothers, an inverse relationship between a calprotectin-positive result and schistosomiasis infection (OR = 0.17. 95% CI = 0.03–0.94, *P* = 0.043) was found. If published cut-off values of 100 μg/g or 150 μg/g were used, there was no association between *S. mansoni* infection and an increased level of fecal calprotectin in children (100 μg/g: *P* = 0.414; 150 μg/g: *P* = 0.598), and only a marginally significant association in mothers (100 μg/g: *P* = 0.162; 150 μg/g: *P* = 0.075).^25^

### Fecal occult blood.

**Figure 3.**
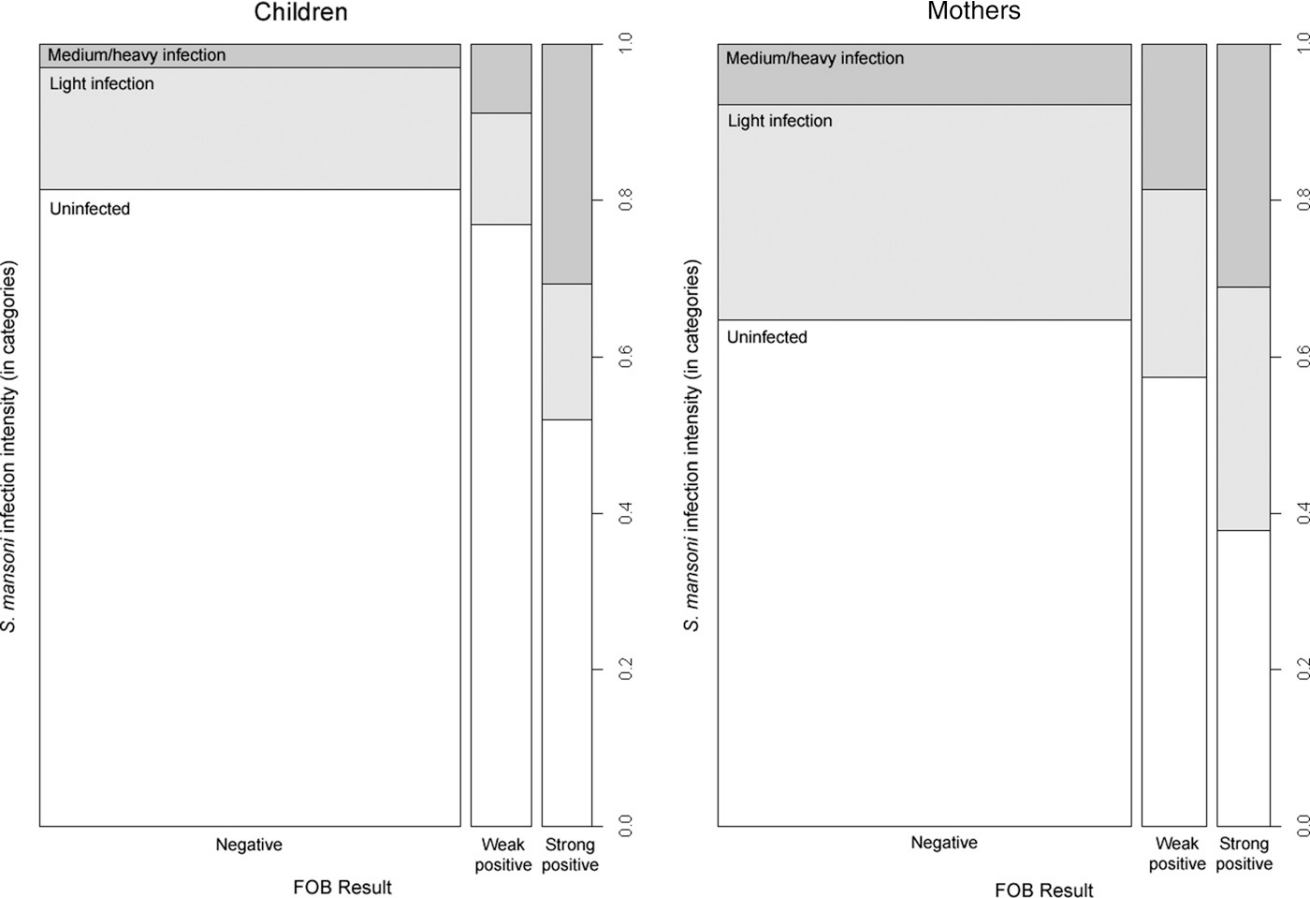
Fecal occult blood (FOB) (negative, weak positive, and strong positive) and *Schistosoma mansoni* infection intensity (negative, light infection, and medium/heavy infection), Uganda. Column widths represent a ratio of 80:11:10 of children with negative, weak positive, and strong positive FOB results and a ratio of 39:6:5 of mothers with negative, weak positive, and strong positive FOB results.

The FOB tests were conducted for 814 children and 448 mothers. On the basis of the results of this test, 36.0% of children and 40.9% of mothers had detectable blood in their stool. When FOB test results were plotted against *S. mansoni* infection intensity, there was an obvious positive correlation for children and mothers (Figure 3). Logistic regression analysis showed that there was a strong association between FOB and schistosomiasis infection in children (OR = 2.30, 95% CI = 1.57–3.35, *P* < 0.0001) and mothers (OR = 1.95. 95% CI = 1.24–3.07, *P* = 0.004), and heavier *S. mansoni* infections were more strongly associated with FOB (Table 3). When data were analyzed separately for each lake, FOB was associated with schistosomiasis infection in children near Lake Albert (OR = 3.27, 95% CI = 1.43–7.43, *P* = 0.005) and Lake Victoria (OR = 1.75, 95% CI = 1.07–2.87, *P* = 0.025) but only in mothers near Lake Victoria (OR = 2.31, 95% CI = 1.39–3.85, *P* = 0.001). In contrast, there was no association between FOB and hookworm infection (Table 3).

In an attempt to further explore the association between FOB test results and schistosomiasis infection, we combined questionnaire results with FOB results and the relationship with schistosomiasis was investigated. In children and mothers, there was no improvement in the association, although in some instances, the OR increased slightly. This finding was accompanied by an expansion of the CIs.

## Discussion

We report the results of an investigation designed to identify markers that could be used in a field situation to assess morbidity associated with intestinal schistosomiasis in young children and mothers in Uganda. These age classes potentially represent early and late stages of morbidity. We found that fecal calprotectin is not a suitable morbidity indicator for intestinal schistosomiasis. Conversely, a self-reported history of blood in stool or diarrhea showed a positive association with schistosomiasis infection in children. In addition, FOB tests show particular promise, especially for assessment of morbidity in young children.

A number of studies have investigated whether questionnaires enquiring about current or past experience of symptoms such as blood in stool and diarrhea can be used for individual assessment of morbidity or to determine which communities are at high risk of disease. Overall blood in stool has been found to be most strongly associated with schistosomiasis infection but the results were variable with a diagnostic sensitivity of 7–66% and specificity of 54–96%.^21^ We found that a history of blood in stool or diarrhea was associated with *S. mansoni* infection in children but not in mothers. However, if children near the two lakes were considered separately, the evidence for a relationship between positive questionnaire responses and *S. mansoni* infection was much weaker, indicating that questionnaire data may only be useful for assessing morbidity when studying large numbers of young children. The fact that questionnaire data were not particularly reliable as morbidity indicators is not surprising. Symptoms such as blood in stool and diarrhea are not specific for intestinal schistosomiasis and can be caused by a number of different infections. In addition, the data depend on the mother's recall of her own symptoms and those of her children, which may be imperfect. In contrast, self-reported blood in urine is found to be commonly associated with urinary schistosomiasis, caused by S. *haematobium*, in schistosomiasis-endemic areas.^33^–^35^

No positive association between fecal calprotectin and schistosomiasis infection was observed in children or mothers when we used a range of cut-off values, which indicated that human calprotectin is unlikely to be suitable as a morbidity marker for intestinal schistosomiais. This finding is surprising given the fact that inflammatory processes in the intestines play an important role in schistosomiasis progression, and that MRP8 and MRP14 have been detected in mononuclear cells at the periphery of granulomas in mice infected with *S. mansoni*.^36^ Chronic inflammation associated with *S. mansoni* eggs appears to involve recruitment of eosinophils rather than neutrophils, which may explain why there is no detectable increase in fecal calprotectin in infected persons.^13^

Nine children but no mothers had a calprotectin concentration outside the ranges previously reported in healthy persons.^37^ The increased calprotectin levels observed in some children may reflect underlying intestinal pathologic changes or could lie within the normal range for children from Africa because our study is the first to investigate fecal calprotectin levels in a population in sub-Saharan Africa. Increased calprotectin levels are observed in newborn and breast-feeding infants,^38^,^39^ but because the youngest child with an increased calprotectin level in our study is one year of age, this finding is unlikely to be relevant to our results.

Even if a correlation were seen between fecal calprotectin and schistosomiasis infection, there would be a number of difficulties associated with widespread deployment of the calprotectin ELISA in the field. First, the ELISA is expensive. Taking into account standards, controls and replicates, we determined that the cost per person would be approximately $20. Second, a reliable cold chain for storage of the kit components and an ELISA plate reader are required, thus necessitating an on-site power source. Third, the protocol is relatively complicated for a field-based setting and requires well-trained staff. Recently, rapid tests for fecal calprotectin have been developed, which may provide a viable alternative to ELISAs in field-based studies but are unlikely to be further informative in this context.^40^,^41^

Our data show a strong association between *S. mansoni* infection and FOB in mothers near Lake Victoria and in children near both lakes. A variety of infections and intestinal conditions can also cause occult blood in feces, including parasitic worms and colorectal cancer. Obviously, colorectal cancer is not an issue for young children and is likely to account for only a small fraction of FOB-positive results in mothers. Interestingly, there was no association between hookworm infection and FOB in this cohort, although there was a low prevalence of hookworm infection near Lake Albert and hookworm infection intensities were generally low near Lake Victoria. Consistent with our results, Kanzaria and others found that persons with higher intensity hookworm infections were no more likely to be fecal occult blood positive than those with low intensity infections or no infection, although they were more likely to be anemic.^28^

We found few *Ascaris* and *Trichuris* infections in this study. Previous work has shown that levels of *Strongyloides stercoralis* and *Entamoeba histolytica* in these communities were also low (Sousa-Figueiredo JC, unpublished data).^42^ Of note, when the same FOB tests were used in Zanzibar where prevalence levels of soil-transmitted helminths were moderate to high, the prevalence of FOB-positive results was only 7% in children and 14% in mothers.^43^ Nevertheless, we cannot entirely exclude the possibility that there is another infectious agent present in our study population, which correlates with schistosomiasis infection and FOB. The FOB test used in this survey uses a double-antibody sandwich assay and is specific for human hemoglobin. Thus, test results were not influenced by diet but may have been affected by blood released from other lesions (e.g., hemorrhoids) or through contamination of fecal material with menstrual blood in mothers.

There are several possible explanations for the stronger association between FOB and *S. mansoni* infection in mothers near Lake Victoria compared with those near Lake Albert. First, there is some genotypic partitioning in the *S. mansoni* parasite between Lake Albert and Lake Victoria, which may be associated with differences in the morbidity induced by the parasite.^44^ Second, there are tribal differences in persons near the two lakes, which may lead to differences in immune responses to *Schistosoma* eggs. Third, near Lake Albert, mothers may have other infections which could lead to FOB and mask the relationship with schistosomiasis. Fourth, infection dynamics can influence morbidity. It is known that immune responses to schistosomiasis are down-modulated over time and the number of eggs produced by adult worms can also vary. Thus, more recent infections may produce greater intestinal bleeding than long-standing infections.^13^

The utility of FOB tests for diagnosis or assessment of morbidity associated with intestinal schistosomiasis has been investigated previously but with tests of differing sensitivities. In China, no relationship was observed between FOB and the presence or intensity of *S. japonicum* infection.^45^ In contrast, studies in Brazil and Zimbabwe showed a positive correlation between intensity of *S. mansoni* infection and percentage of FOB-positive stools.^27^,^46^ Similarly, in the Philippines, persons with heavy *S. japonicum* infections were 3.5 times more likely to be FOB positive than those with no or light to moderate infections.^28^ To our knowledge, the present study is the first to use an immunochemistry-based test to assess the association between *S. mansoni* infection and FOB and the first to show a positive relationship between schistosomiasis infection/intensity and FOB in young children.

The FOB test used for this study is well suited to field conditions. All components can be stored at room temperature and the test is rapid and simple, enabling hundreds of stool samples to be tested per day. The only disadvantage is that it is relatively expensive, costing approximately $1.70 per person. Future follow-ups of the cohort will hope to ascertain if FOB can track the dynamics of morbidity after treatment with praziquantel. If the test meets this expectation, it could play an important role in assessing patterns of morbidity reduction associated with ongoing and future national control programs.

Fecal calprotectin was not informative in assessment of the morbidity associated with intestinal schistosomiasis. Although self-reported abdominal symptoms may be of use for screening large populations of young children, they are far less useful on a smaller scale. In contrast, fecal occult blood, as assessed by using an immunochemistry-based rapid test, is strongly associated with prevalence and intensity of intestinal schistosomiasis infection in young children in Uganda. Thus, this test should prove to be a useful tool for community-level determination of intestinal morbidity in young children in sub-Saharan Africa and hopefully for assessment of reductions in morbidity in response to mass chemotherapy.

## Figures and Tables

**Table 1:** Prevalence levels of *Schistosoma mansoni*, STH infections, and morbidity indicators, Uganda*

Characteristic	Organism or factor	Intensity of infection	Children, % (95% CI)	Mothers % (95% CI)
Schistosomiasis	*S. mansoni*	Any	27.2 (24.8–29.7)	47.6 (43.9–51.2)
		Light	18.7 (16.6–20.9)	29.2 (25.9–32.7)
		Medium	6.0 (4.8– 7.5)	12.7 (10.3–15.3)
		Heavy	2.5 (1.7–3.5)	5.7 (4.1–7.7)
STHs	*Ascaris lumbricoides*	Any	0.2 (0.05–0.7)	0.28 (0.03–1.0)
	*Trichuris trichiura*	Any	1.6 (1.0–2.5)	2.2 (1.3–3.6)
	Hookworm	Light	9.8 (8.2–11.6)	30.5 (27.1–34.0)
		Medium/heavy	0.08 (0.002–0.4)	2.1 (1.2–4.4)
Morbidity indicators	Calprotectin	0 mg/g	73.5 (62.7–82.6)	86.2 (74.6–93.9)
		< 150 mg/g	12.1 (5.9–21.0)	5.2 (1.1–14.4)
		> 150 mg/g	14.5 (7.7–23.9)	8.6 (2.9–19.0)
	FOB	–	64.0 (60.6–67.3)	59.2 (54.4–63.7)
		Trace	15.2 (12.8–17.9)	18.8 (15.2–22.7)
		+	11.4 (9.3–13.8)	12.1 (9.2–15.4)
		++	5.5 (4.1–7.3)	6.3 (4.2–8.9)
		+++	3.8 (2.6–5.4)	3.8 (2.2–6.0)

* CI = confidence interval determined using the exact method^47^; STH = soil-transmitted helminthes; FOB = fecal occult blood.

**Table 2:** Association between questionnaire responses and *Schistosoma mansoni* infection in children, Uganda*

Response	No.	% Positive*	OR (95% confidence interval)	*P*
Blood in stool	1,169	43.7	2.06 (1.59–2.67)	< 0.0001
Diarrhea	1,238	78.7	2.55 (1.12–2.43)	0.011
Abdominal pain	1,286	70.5	1.35 (0.98–1.85)	0.066
Blood in stool and diarrhea and abdominal pain	1,119	35.2	1.43 (1.07–1.93)	0.017

* Percentage of persons who reported a history of the relevant sign or symptom.

**Table 3:** Association between FOB and *Schistosoma mansoni* infection, Uganda*

Infection	Children				Mothers			
	No.	% FOB positive	OR (95% CI)	*P*†	No.	% FOB positive	OR (95% CI)	*P*†
*S. mansoni*								
Negative	627	17.4	1.00 (–)	–	274	17.5	1.00 (–)	–
Positive	175	32.6	2.30 (1.57–3.35)	< 0.0001	174	29.3	1.95 (1.24–3.07)	0.004
Light infection	125	20.8	1.25 (0.77–2.01)	0.364	123	22.0	1.32 (0.78–2.25)	0.306
Medium infection	36	52.8	5.31 (2.67–10.6)	< 0.0001	37	51.3	4.96 (2.41–10.2)	< 0.0001
Heavy infection	14	85.7	28.5 (6.29–129)	< 0.0001	14	35.7	2.52 (0.80–7.92)	0.115
Hookworm								
Negative	690	20.9	1.00 (–)	–	241	22.0	1.00 (–)	–
Positive	112	19.6	0.93 (0.56–1.53)	0.776	207	22.2	1.01 (0.65–1.59)	0.953

* FOB = fecal occult blood; OR = odds ratio; CI = confidence interval.

† By Wald test.
